# Estimation of standing height in spina bifida: model development and validation

**DOI:** 10.1016/j.jped.2024.06.005

**Published:** 2024-07-16

**Authors:** Fabio Bertapelli, Marisa Maia Leonardi-Figueiredo, Emanuela Juvenal Martins, Cyntia Rogean de Jesus Alves de Baptista, Ana Claudia Mattiello-Sverzut

**Affiliations:** Faculdade de Medicina de Ribeirão Preto, Departamento de Ciências da Saúde, Universidade de São Paulo, Ribeirão Preto, SP, Brazil

**Keywords:** Linear growth, Arm span, Height, Spina bifida, Children, Adolescents

## Abstract

**Objective:**

Childhood standing height has been estimated from arm span-related (height_AS_) models. The authors aimed to develop and cross-validate a height_AS_ model in individuals with spina bifida (SB) and examine the accuracy of existing height_AS_ models.

**Methods:**

Participants were individuals with sacral and low-lumbar SB (*n* = 14) and non-SB (*n* = 83), 7–16 years old. Arm span, age, sex, and group (SB vs. non-SB) were candidate height predictors. Sequential regression and leave-one-out cross-validation approaches were used for the model development (M1) and cross-validation (M1–M5). Existing models were: an SB-specific model from Polfuss et al. (M2) and non-SB specific models from Gauld et al. (M3), Mulu et al. (M4), and Zverev et al. (M5) studies.

**Results:**

Arm span and group explained 95 % of the variance in height (R^2^ = 0.95; *p* < 0.001; SEE = 3.666 cm) and were included in the M1. Mean differences between actual and estimated height were 0.0 cm (M1), 0.4 cm (M2), and 0.5 cm (M5), all not significant (*p* > 0.05). However, Bland-Altman analysis revealed some variability in the predictability of the models across participants with limits of agreement ranging from 7.4 to 10.9 cm. Considerable errors were observed with M3 (mean diff: −5.58 cm, 95 % CI: −1.6, −20.2 cm), and M4 (mean diff: 10.5 cm, 95 % CI: −13.8, −27.3 cm).

**Conclusions:**

Models (M1, M2 and M5) may accurately estimate standing height in groups of children with SB. However, due to the wide limits of agreement, caution is recommended when applying these models for individual height estimations.

## Introduction

Spina bifida (SB), a condition characterized by a neural tube defect, is associated with significant limitations in neuromotor and sensory systems.^1^ With an estimated population prevalence of 34–81 per 100,000 live births,^2^ it has been demonstrated that children and adolescents with SB experience several physical and cognitive conditions such as weakness or paralysis of the lower limbs,^1^ lower physical fitness,^3^ reduced physical activity levels,^4^ sleep disorders,^5^ and impaired executive functions and attention^6^ compared with their peers without disabilities. It has further been demonstrated that this population is at higher risk for growth faltering than the general population. For example, previous studies have observed that participants with SB aged 3–14 years had lower height based on sex- and age-specific growth references.^7^, ^8^, ^9^ Since linear growth is the best overall indicator of childhood well-being,^10^ measuring the height in children and adolescents with SB is a priority for health research and clinical practice.

Standing height is a well-recognized measure of linear growth in school-aged children and adolescents.^11^ This measure, however, cannot be easily obtained from children and adolescents with SB who have weakness or paralysis of the lower limbs. Recumbent length is another measure for monitoring linear growth, but it can still be inappropriate due to common lower limb contractures associated with neuromuscular diseases.^12^ Therefore, alternative anthropometrics for estimating the standing height, which would allow for easy application to large cohorts of children and adolescents with SB, are required.

An approach that can be easily standardized and applied within population-level linear growth monitoring involves measuring the arm span. It has been observed that arm span predicts standing height among children and adolescents without disabilities.^13^, ^14^, ^15^, ^16^, ^17^ Past research has developed and cross-validated models for estimating standing height based on arm span (height_AS_) in children and adolescents with and without disabilities.^15^, ^16^, ^17^, ^18^ Polfuss et al.^18^ developed an SB-specific standing height model that includes arm span, age, and lesion levels (sacral, lumbar, and thoracic) in a group of 418 children and adolescents with SB from the United States. In contrast, the models developed by Gauld et al.^17^ and Zverev et al.^15^ were non-SB specific and included arm span and age, while the model from Mulu et al. was non-SB specific and included only arm span. Since the models by Gauld et al.,^17^ Zverev et al.,^15^ and Mulu et al.^16^ were developed for children without SB, the accuracy in estimating standing height in children and adolescents with SB remains uninvestigated. Considering that children and adolescents with SB are at a higher risk for lower height compared to their peers without SB,^7^, ^8^, ^9^ it is hypothesized that existing height estimation models may be biased for individuals with SB. The model by Polfuss et al.^18^ is anticipated to perform better in estimating standing height, as it was specifically developed for children and adolescents with SB, although it has not yet been validated for estimating linear growth in this population.

Therefore, there is a need to develop and cross-validate a height_AS_ model to support health professionals and researchers for population-level linear growth surveillance in this population. Additionally, it is important to compare the performance of a specific height_AS_ model with existing height_AS_ models. The aim of this study was to develop and cross-validate a height_AS_ model for estimating standing height in children and adolescents with SB. This study further aimed to examine the performance of an SB specific model from Polfuss et al.^18^ and non-SB specific models from Gauld et al.,^17^ Mulu et al.,^16^ and Zverev et al.,^15^ for estimating standing height in children and adolescents with SB.

## Methods

### Participants

The authors recruited participants with and without SB at the Rehabilitation Center at the University of São Paulo, Ribeirão Preto, Brazil, and the surrounding communities. Inclusion criteria for this study were: 1) participants with and without SB with ages following the school-aged World Health Organization definition (i.e., 5–19 years old); 2) participants who were ambulatory and did not have any orthopedic conditions that could affect the measurement of height and horizontally outstretched arms span; and 3) ability to understand procedures. The protocol of this study was approved by the ethics committee of the Ribeirão Preto Medical School, University of São Paulo, with the Declaration of Helsinki followed during all study procedures. The authors obtained written informed consent from all participants and their parents/guardians. The authors included a total of 83 participants without SB (46 males and 37 females; ages 12.13 ± 2.75 years) and 14 participants with SB (5 males and 9 females; ages 10.51 ± 2.23 years). All participants with SB had spinal cord injury at the sacral (*n* = 11) and low lumbar (*n* = 3) levels.

### Anthropometric measures

Standing height and arm span were obtained from all participants by experienced technicians following standardized procedures. Standing height was measured with a stadiometer to the nearest 0.1 cm. Arm span was measured as the distance between the tips of the middle fingers with arms outstretched horizontally using a metal tape to the nearest 0.1 cm, with participants in a seated position.

### Data analyses

Data analyses were performed in SPSS Statistics 22 (IBM, Armonk, NY). The alpha level was set at 0.05. The normality of data was examined using the Shapiro–Wilk test, histograms, boxplots, and Q-Q-plots. Comparisons of demographic and anthropometric variables between participants with and without SB were examined with an independent-sample t-test and Mann-Whitney U test. Spearman rho rank-order correlation (r_s_) was used to examine the bivariate association between height and arm span. The height prediction model was developed using sequential multiple regression. Independent variables were initially entered in the regression following this step sequence: 1) arm span; 2) age; 3) sex; and 4) group (SB vs. non-SB). The coefficient of determination (R^2^) and standard error of estimate (SEE) were used to examine the goodness-of-fit of the final model. The present height estimation model was cross-validated using the leave-one-out cross-validation approach. Specifically, this approach involved splitting the whole sample (*n* = 97) into training samples (i.e., all participants except one; *n* = 96) and validation samples (i.e., the omitted participant; *n* = 1). This process was made in two steps: in step 1, the authors ran a model (model 1) using a first training sample (sample 1a; participant ID 2, 3…97); and in step 2, the authors used the resulting regression coefficients from the model 1 to estimate the height in the first validation sample (sample 1b; participant ID 1). The authors repeated steps 1–2 involving the resting modeling process for all possible subsamples of 97 participants. Agreement between actual and estimated height was examined with mean absolute error (MAE), root mean square error (RMSE), and Bland-Altman Analysis. Additionally, the Pearson correlation coefficient (r) was used to examine the associations between actual and estimated height. The square of this Pearson correlation coefficient was compared to the R^2^ of the original regression model for the evaluation of the generalizability of the regression model as previously recommended.^19^ Moreover, the authors compared the actual and estimated mean height with paired samples *t*-test.

Additionally, the authors estimated the height of the present participants with SB using an SB specific model from Polfuss et al.^18^ (M2_Polfuss et al._) and three non-SB specific models from Gauld et al.^17^ (M3_Gauld et al._), Mulu et al.^16^ (M4_Mulu et al._), and Zverev et al.^15^ (M5_Zverev et al._):

M2_Polfuss et al._^18^

$$\begin{array}{c} \text{Sacral}\;\text{level}\;\text{SB}:\text{height}=20.2+\left(0.47\times \text{age}\right)+\left(0.80\times \text{arm}\;\text{span}\right)\\ \text{Low}‐\text{lumbar}\;\text{level}\;\text{SB}:\text{height}=20.2+\left(0.47\times \text{age}\right)+\;\left(0.80\times \text{arm}\;\text{span}\right)-3.60 \end{array}$$

M3_Gauld et al._^17^

(1) $$\begin{array}{c} \text{Boys}:\text{height}=16.258+\left(0.829\times \text{arm}\;\text{span}\right)+\left(0.721\times \text{age}\right)\\ \text{Girls}:\text{height}=36.976+\left(0.619\times \text{arm}\;\text{span}\right)+\left(1.593\times \text{age}\right) \end{array}$$

M4_Mulu et al._^16^

(2) $$\begin{array}{c} \text{Boys}:\text{height}=33.11+\left(0.792\times \text{arm}\;\text{span}\right)\\ \text{Girls}:\text{height}=62.59+\left(0.597\times \text{arm}\;\text{span}\right) \end{array}$$

M5_Zverev et al._^15^

(3) $$\begin{array}{c} \text{Boys}:\text{height}=17.043+\left(0.348\times \text{age}\right)+\left(0.815\times \text{arm}\;\text{span}\right)\\ \text{Girls}:\text{height}=18.158+\left(0.265\times \text{age}\right)+\left(0.817\times \text{arm}\;\text{span}\right) \end{array}$$

For all models, arm span is in cm and age is in years.

The performance of the height estimated by these models was evaluated with MAE, RMSE, and Bland–Altman analysis.

## Results

Table 1 presents the age and anthropometric characteristics of the participants with and without SB. There were significantly lower age, height, and arm span in participants with SB than in non-SB (*p* ≤ 0.05; Table 1).

**Table 1:** Age and anthropometric characteristics of children and adolescents with and without spina bifida.

Characteristics	SB (*n* = 14)		Non-SB (*n* = 83)		*p*
	Mean	SD	Mean	SD	
Age (years)	10.51	2.23	12.13	2.75	0.044^b^⁎
Height (cm)	138.43	12.66	153.59	15.21	0.001^a^†
Arm span (cm)	144.64	16.84	155.39	16.36	0.045^b^⁎

Note: data are presented as mean and standard deviation (SD), and frequencies (n); SB, spina bifida; non-SB, without spina bifida.

a Independent t-test.

b Mann-Whitney U test.

⁎ p <0.05.

† p < 0.01.

Significant bivariate correlation was observed between actual height and arm span in overall (r_s_ = 0.96; *p* < 0.001), non-SB (r_s_ = 0.97; *p* < 0.001), and SB (r_s_ = 0.93; *p* < 0.001) groups. Sequential regression indicated that arm span was a significant predictor of height (R^2^ = 0.93; *p* < 0.001; SEE = 4.150 cm). There were no additional contributions of age (ΔR^2^ = 0.002; *p* = 0.083) and sex (ΔR^2^ = 0.000; *p* = 0.459) to the model. Group (SB vs. non-SB) had an additional contribution to the height (ΔR^2^ = 0.016; *p* < 0.001). Therefore, the final model (M1_Present_) included arm span and group (R^2^ = 0.95; *p* < 0.001; SEE = 3.666 cm). Unstandardized regression coefficients are presented in Table 2. The M1_Present_ for the estimation of standing height in participants with and without SB was:

**Table 2:** Regression model for the estimation of height based on arm span and group (SB vs. non-SB).

Predictor	Unstandardized Regression coefficients		
	b	SE	*p*
Intercept	17.091	3.580	<0.001^a^
Arm span	0.878	0.023	<0.001^a^
Group	−5.722	1.088	<0.001^a^

Note: b, unstandardized coefficient; SE, standard error.

a *p* < 0.001.

M1_Present_:

(4) Height=17.091+(0.878×armspan)−(5.722×group)

For this model, the arm span is in cm, and the group is 0 = non-SB and 1 = SB.

**Figure 1:**
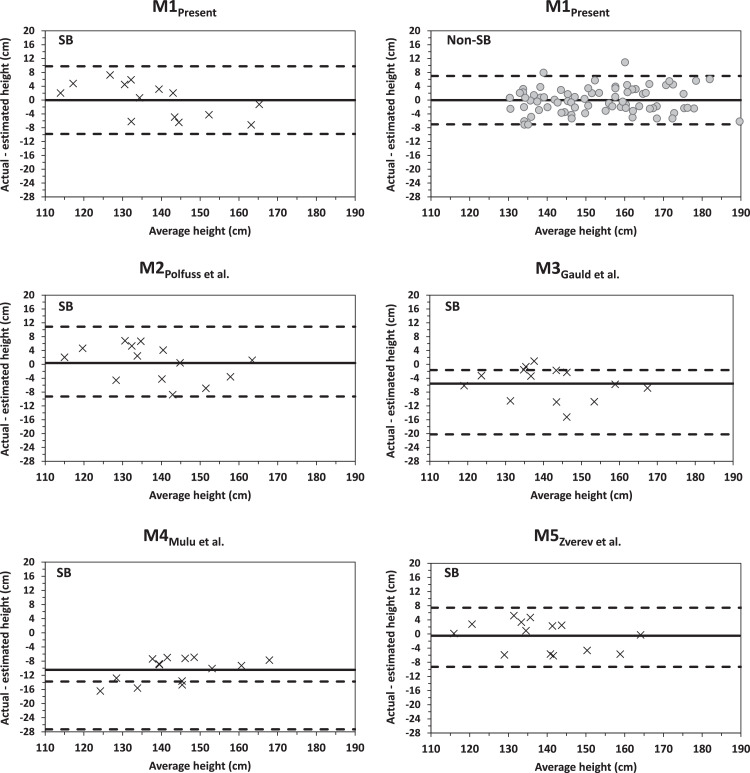
SB, participants with spina bifida; Non-SB, participants without spina bifida; Bland–Altman plot of the difference between actual and estimated height (y-axis) against their average (x-axis) using the M1_Present_ - the model developed in this study, M2_Polfuss et al._ - the model from Polfuss et al.,^18^ M3_Gauld et al._ - the model from Gauld et al.,^17^ M4_Mulu et al._ - the model from Mulu et al.,^16^ and M5_Zverev et al._ - the model from Zverev et al.^15^ among participants with SB (cross marker) and without spina bifida (grey circle marker); dashed lines show 95 % limits of agreement; solid line represents the mean difference.

The M1_Present_ was cross-validated based on strong association and non-significant differences between actual height and estimated height_AS_, as well as small MAE, RMSE, and differences in Bland–Altman analysis (Table 3 and Figure 1). Specifically, the authors observed a strong significant association between actual and height_AS_ estimates in the SB group (Table 3 = 0.95; *p* < 0.001). The high generalizability of the model was indicated by small differences between the square of this Pearson correlation coefficient (0.90) with the coefficient of determination of the model (0.95) (Table 3). Differences between actual height and estimated height_AS_ in the SB group were not significant (actual height: 138.43 ± 12.66 cm; and estimated height_AS_: 138.44 ± 15.05 cm; *p* = 0.993) (Table 3). MAE was 4.32 ± 2.20 cm and RMSE was 1.83 cm (Table 3). In the Bland–Altman plot, the mean difference between actual height and estimated height_AS_ was close to zero (mean error: −0.01 cm; 95 % CI: 9.76, −9.81 cm; Figure 1, and Table 3).

**Table 3:** Performance of arm span-related models for the estimation of height among participants with and without SB of the present study.

Group	Model	Mean (SD)^a^	MAE^a^	RMSE^a^	Mean diff (95 % CI)^a^^,^^b^	r	r^2^
Non-SB	Actual	153.59 (15.21)	–	–	–	–	–
Non-SB	M1_Present_	153.60 (14.37)	2.92	3.28	−0.01 (7.00, −7.00)	0.97^d^	0.94
SB	Actual	138.43 (12.66)	–	–	–	–	–
SB	M1_Present_	138.44 (15.05)	4.32	1.83	−0.01 (9.76, −9.81)	0.95^d^	0.90
SB	M2_Polfuss et al._	138.03 (14.77)	4.41	1.89	0.40 (10.87, −9.29)	0.94^d^	0.88
SB	M3_Gauld et al._	144.01 (13.84)^c^	5.72	2.74	−5.58 (−1.63, −20.25)	0.94^d^	0.88
SB	M4_Mulu et al._	148.90 (10.93)^c^	10.47	4.17	−10.47 (−13.77, −27.28)	0.97^d^	0.94
SB	M5_Zverev et al._	138.91 (14.14)	3.58	1.57	−0.48 (7.42, −9.31)	0.96^d^	0.92

Note: SB, participants with spina bifida; Non-SB, participants without spina bifida; MAE, mean absolute error; RMSE, root mean square error.

a values are in cm.

b mean difference and 95 % confidence intervals from Bland–Altman analysis.

c significance (*p* < 0.01) between estimated and actual height in paired *t*-tests.

d significance (*p* < 0.001) for Pearson correlations between actual and estimated height; M1_Present_ - the model developed in this study, M2_Polfuss et al._ - the model from Polfuss et al.,^18^ M3_Gauld et al._ - the model from Gauld et al.,^17^ M4_Mulu et al._ - the model from Mulu et al.,^16^ and M5_Zverev et al._ - the model from Zverev et al.^15^

Predictive performance results for previously published height_AS_ models are presented in Table 3 and Figure 1. The authors observed that the M2_Polfuss et al._ and M5_Zverev et al._ models had better predictive performance for the estimation of standing height compared to M3_Gauld et al._ and M4_Mulu et al._ models. Specifically, the authors observed that differences between actual and estimated height were significant using M3_Gauld et al._ (mean diff: −5.58 cm, *p* < 0.01) and M4_Mulu et al._ (mean diff: −10.47 cm, *p* < 0.01) models and non-significant using M2_Polfuss et al._ (mean diff: −0.40 cm, *p* = 0.775) and M5_Zverev et al._ (mean diff: −0.48 cm, *p* = 0.679) models (Table 3). Moreover, M5_Zverev et al._ model had narrower limits of agreement (M5_Zverev et al._ 95 % CI: 7.42, −9.31 cm) compared to other models in Bland-Altman analysis (Table 3 and Figure 1).

## Discussion

This study developed and cross-validated a height_AS_ model for the estimation of standing height among children and adolescents with SB. This study further observed that M2_Polfuss et al._ (SB-specific model) and M5_Zverev et al._ (non-SB specific model) had the similar predictive ability to estimate standing height. Developed and validated specific prediction models would be useful for researchers in estimating standing height among children and adolescents with SB.

The present study demonstrated that arm span had a strong bivariate association with height among participants with SB (*r* = 0.93) and without SB (*r* = 0.97). The present observations are consistent with previous data that indicated that arm span was moderate and strongly correlated with height (*r* = 0.71 to 0.98) among children and adolescents aged 7–18 years without disabilities.^13^, ^14^, ^15^, ^16^ Moreover, this study observed that arm span alone explained 93 % of the variance in height in the present sample. A finding that should be considered, however, is that arm span alone did not explain 7 % of the variance in standing height in this study. There is evidence that age and sex are additional significant predictors of height among children and adolescents from the general population. However, the authors found no contributions of these variables to this study's model, necessitating an examination of other potential predictors. In the present study, the authors observed that the group (SB vs. non-SB) explained an additional 2 % of the variance in standing height. The effect of the group was expected given that children and adolescents with SB are at higher risk for short height.^7^, ^8^, ^9^ Taken together, arm span and group were significant predictors of height and were therefore included in the final model for the estimation of standing height. The next step involved cross-validating this arm span-related model.

The authors provided evidence that the M1_Present_ was valid for estimating standing height in the present participants with and without SB. Errors based on MAE and RMSE were relatively small in the whole cross-validation process. Moreover, there was a high generalizability of the model as indicated by minimal differences in the associations between training and validation samples. Additionally, the authors observed non-significant differences between actual and estimated height using this model. These findings agree with a previous cross-validation study that indicated non-significant mean differences and strong associations between actual height and estimated height_AS_ among Malawians aged 6–15 years (the M5_Zverev et al._ model).^15^ Another important finding was that the model produced a zero mean difference and somewhat wide 95 % limits of agreement in Bland-Altman analysis.

Another important finding in this study was that the M1_Present_ model had a similar predictive ability in comparison with an SB specific (M2_Polfuss et al._) and a non-SB specific (M5_Zverev et al._) model for standing height estimation of children and adolescents with SB. A closer inspection of the M1_Present_ and M2_Polfuss et al._ Bland-Altman plots, however, indicate a trend from overestimation at lower average heights to underestimation at higher average heights. Moreover, the authors observed that M5_Zverev et al._ model had a minimal trend and smaller MAE, RMSE, and 95 % limits of agreement in Bland–Altman analysis compared to M1_Present_ and M2_Polfuss et al._ models. A consideration is that the M5_Zverev et al._ model was derived from a sample with somewhat small differences in anthropometrics when compared to this sample with SB. For example, the authors found that the present sample had mean arm span and height z-scores varying from −0.6 to −1.5 based on the data from the Zverev study. It was further observed considerable mean height differences between actual, M3_Gauld et al.,_ and M4_Mulu et al._ models, with limits of agreement reaching −27 cm. A possible explanation for large differences between M3_Gauld et al.,_ and M4_Mulu et al._ models may include variations in age, height, and arm span between samples. It is additionally important to note that M3_Gauld et al._ and M5_Zverev et al._ models included age in the sex-specific models. By comparison, the authors observed that age had no contributions to the present regression model after accounting for the effect of arm span. Yet, the development of sex-specific models was not possible in this study because the sample size was not adequate to capture the influence of the group – future research is needed to address this issue. Another important consideration is that existing height_AS_ models evaluated in this study were developed in children and adolescents from the United States, Ethiopia, Africa, and Australia. Research has demonstrated that linear growth, especially height, varies considerably in children and adolescents from different geographical regions.^20^

Measuring standing height from the height_AS_ has implications for research and clinical practice. Taken together, M1_Present_, M2_Polfuss et al._, and M5_Zverev et al._ models may be used with confidence in research contexts, as these models demonstrated minimal errors on a group basis, but caution regarding sample profiles and measurement protocols is required. Applying these models would support researchers in evaluating linear growth in participants with SB with posture difficulties due to weakness or paralysis of the lower limbs. The length of body segments has been widely used for measuring height among individuals with physical disabilities.^21^ Additionally, the present model may be useful for supporting large-scale growth surveillance research among participants with SB. Past research has demonstrated that children and adolescents with SB may be at high risk for short height.^7^, ^8^, ^9^ It is recognized that height is a fundamental predictor of overall health in children and adolescents.^10^ However, due to the wide limits of agreement, caution is advised when using M1_Present_, M2_Polfuss et al._, and M5_Zverev et al._ models for the evaluation of linear growth in clinical practice, as these models may not demonstrate adequate predictive performance on an individual basis. Moreover, future research with large sample sizes is necessary to test the ability of the presented height_AS_ models to identify individuals with SB presenting with below- and above-average standing height based on the interpolation using sex- and age-specific values from growth references such as those from Center from Centers for Disease Control and Prevention and the World Health Organization. Additionally, M3_Gauld et al.,_ and M4_Mulu et al._ models are not recommended for clinical and research purposes, as the differences observed were beyond the acceptable limits of agreement.

This study has some limitations and strengths that should be considered. Limitations include cross-sectional data from a small sample of participants with SB. Additionally, there were no participants with spinal cord injury at mid- and high-lumbar, and thoracic levels. This study also focused on a narrow age range of individuals with spina bifida (7–16 years old), limiting the applicability of the findings to those outside this age group. Finally, although this model was validated based on leave-one-out cross-validation approach, future research should consider cross-validating the model in other samples. Strengths of this study include measuring height in a standing position. This would allow comparison of standing height data of children with SB with growth standards following standardized procedures. Moreover, the authors cross-validated the present model including two simple variables and examined the performance of existing SB and non-SB specific models, enabling the practical application of the models in epidemiological research contexts.

This study indicated that arm span was strongly associated with standing height in children and adolescents with SB. A model including arm span and group was developed and cross-validated for estimating standing height in individuals with sacral and low-lumbar SB aged 7–16 years. Existing models (M2_Polfuss et al._, and M5_Zverev et al._) had similar height predictability compared to the present model (M1_Present_). These models may be useful to researchers for linear growth surveillance in children and adolescents with SB. However, due to the wide limits of agreement, the authors recommend caution if applying these models for individual estimation of standing height.

## Funding

This study was supported by “São Paulo Research Foundation (FAPESP)” [grant numbers: 2017/17596-4; 2022/00099-6]; “Coordenação de Aperfeiçoamento de Pessoal de Nível Superior” (Capes) [scholarship numbers: 88887.586702/2020-00; 001] and “Fundação de Apoio ao Ensino, Pesquisa e Assistência do Hospital das Clínicas da Faculdade de Medicina de Ribeirão Preto da Universidade de São Paulo” (FAEPA).

## Conflicts of interest

The authors declare no conflicts of interest.

## References

[1] Copp A.J., Adzick N.S., Chitty L.S., Fletcher J.M., Holmbeck G.N., Shaw G.M. (2015). Spina bifida. Nat Rev Dis Prim.

[2] Atta C.A., Fiest K.M., Frolkis A.D., Jette N., Pringsheim T., St Germaine-Smith C. (2016). Global birth prevalence of spina bifida by folic acid fortification status: a systematic review and meta-analysis. Am J Public Health.

[3] Leonardi-Figueiredo M.M., de Queiroz Davoli G.B., Avi A.E., Crescêncio J.C., Moura-Tonello S.C., Manso P.H. (2021). Cardiac autonomic modulation of heart rate recovery in children with spina bifida. Int J Sports Med.

[4] Claridge E.A., Bloemen M.A., Rook R.A., Obeid J., Timmons B.W., Takken T. (2019). Physical activity and sedentary behaviour in children with spina bifida. Dev Med Child Neurol.

[5] Murray C.B., Palermo T.M., Holmbeck G.N. (2018). A multimethod, case-controlled study of sleep-wake disturbances in adolescents with spina bifida. J Pediatr Psychol.

[6] Rose B.M., Holmbeck G.N. (2007). Attention and executive functions in adolescents with spina bifida. J Pediatr Psychol.

[7] Greene S.A., Frank M., Zachmann M., Prader A (1985). Growth and sexual development in children with meningomyleocoele. Eur J Pediatr.

[8] Trollmann R., Strehl E., Wenzel D., Dörr H.G. (1998). Arm span, serum IGF-1 and IGFBP-3 levels as screening parameters for the diagnosing of growth hormone deficiency in patients with myelomeningocele - preliminary data. Eur J Pediatr.

[9] Wren T.A., Mueske N.M., Rethlefsen S.A., Kay R.M., Van Speybroeck A., Mack W.J. (2020). Quantitative computed tomography assessment of bone deficits in ambulatory children and adolescents with spina bifida: importance of puberty. JBMR Plus.

[10] de Onis M., Branca F. (2016). Childhood stunting: a global perspective. Matern Child Nutr.

[11] de Onis M., Onyango A.W., Borghi E., Siyam A., Nishida C., Siekmann J. (2007). Development of a WHO growth reference for school-aged children and adolescents. Bull World Health Org.

[12] Skalsky A.J., McDonald C.M. (2012). Prevention and management of limb contractures in neuromuscular diseases. Phys Med Rehabil Clin N Am.

[13] Yabanci N., Kiliç S., Şimşek I. (2010). The relationship between height and arm span, mid-upper arm and waist circumferences in children. Ann Hum Biol.

[14] Monyeki K.D., Sekhotha M.M. (2016). The relationships between height and arm span, mid-upper arm and waist circumferences and sum of four skinfolds in Ellisras rural children aged 8–18 years. Public Health Nutr.

[15] Zverev Y., Chisi J. (2005). Estimating height from arm span measurement in Malawian children. Coll Antropol.

[16] Mulu A., Sisay B. (2021). Estimation of stature from arm span, arm length and tibial length among adolescents of aged 15–18 in Addis Ababa, Ethiopia. Ethiop J Health Sci.

[17] Gauld L.M., Kappers J., Carlin J.B., Robertson C.F. (2004). Height prediction from ulna length. Dev Med Child Neurol.

[18] Polfuss M., Liu T., Smith K., Murphy P.S., Ward E., Thibadeau J. (2022). Weight status of children participating in the national spina bifida patient registry. Pediatrics.

[19] Tabachnick B.G., Fidell L.S. (2019).

[20] Rodriguez-Martinez A., Zhou B., Sophiea M.K., Bentham J., Paciorek C.J., Iurilli M.L. (2020). Height and body-mass index trajectories of school-aged children and adolescents from 1985 to 2019 in 200 countries and territories: a pooled analysis of 2181 population-based studies with 65 million participants. Lancet.

[21] Bragança S., Castellucci I., Costa E., Arezes P., Carvalho M. (2020). Anthropometric data for wheelchair users: a systematic literature review. Int J Occup Saf Ergon.

